# Carbon disulfide removal from gasoline fraction using zinc-carbon composite synthesized using microwave-assisted homogenous precipitation

**DOI:** 10.1007/s11356-023-27905-2

**Published:** 2023-06-14

**Authors:** Ayat A.-E. Sakr, Nouran Amr, Mohamed Bakry, Waleed I. M. El-Azab, Mohamed A. Ebiad

**Affiliations:** 1 Analysis & Evaluation Division, Egyptian Petroleum Research Institute, Nasr City, Cairo, 11727 Egypt; 2 Faculty of Biotechnology, October University for Modern Sciences and Arts (MSA), Giza, Egypt

**Keywords:** Clean fuel, Carbon disulfide, Zinc-carbon composite, Urea hydrolysis, Biomass, Adsorption kinetics

## Abstract

Carbon disulfide (CS_2_) is one of the sulfur components that are naturally present in petroleum fractions. Its presence causes corrosion issues in the fuel facilities and deactivates the catalysts in the petrochemical processes. It is a hazardous component that negatively impacts the environment and public health due to its toxicity. This study used zinc-carbon (ZC) composite as a CS_2_ adsorbent from the gasoline fraction model component. The carbon is derived from date stone biomass. The ZC composite was prepared via a homogenous precipitation process by urea hydrolysis. The physicochemical properties of the prepared adsorbent are characterized using different techniques. The results confirm the loading of zinc oxide/hydroxide carbonate and urea-derived species on the carbon surface. The results were compared by the parent samples, raw carbon, and zinc hydroxide prepared by conventional and homogeneous precipitation. The CS_2_ adsorption process was performed using a batch system at atmospheric pressure. The effects of adsorbent dosage and adsorption temperatures have been examined. The results indicate that ZC has the highest CS_2_ adsorption capacity (124.3 mg.g^−1^ at 30 °C) compared to the parent adsorbents and the previously reported data. The kinetics and thermodynamic calculation results indicate the spontaneity and feasibility of the CS_2_ adsorption process.

**Supplementary Information:**

The online version contains supplementary material available at 10.1007/s11356-023-27905-2.

## Introduction

According to EIA (2021) and BP (2022), liquid petroleum fuels are considered the largest source of energy (EIA 2021; BP 2022). Petroleum or crude oil is composed mainly of hydrocarbon and may contain heteroatoms such as sulfur, oxygen, nitrogen, and metals. The type of crude oil can be classified according to the sulfur content, whether sweet or sour. Sour crude oil if it contains total sulfur of more than 0.5 wt%. Sulfur components have a corrosive action on pipelines, pumping, and refining equipment. Also, it deactivates the catalysts during the refining processes (Kohl and Nielsen 1997; Hsu and Robinson 2017; Saleh 2020; Bhargava et al. 2022). Different forms of sulfur species may be present in petroleum which vary according to their origin, such as hydrogen sulfide (H_2_S), carbonyl sulfide (COS), carbon disulfide (CS_2_), mercaptans, sulfides, and thiophenes (Stumpf et al. 1998; Han et al. 2018; Saleh 2020).

Carbon disulfide (CS_2_) is a type of sulfur component that can be present naturally in petroleum fractions such as gasoline (Stumpf et al. 1998; Rhodes et al. 2000; Yi et al. 2014). It is a non-polar linear molecule. In the pure state, CS_2_ is a colorless liquid with a pleasant smell; however, it has a pale yellow color with an offensive odor if it is impure (Bocos-Bintintan and Ratiu 2020).

CS_2_ has many industrial applications, such as manufacturing viscous rayon, cellophane films, rubber, carbon tetrachloride, xanthates, thiourea, and mercaptans. It is a powerful solvent for materials such as resins, fates, rubbers, fertilizers, etc. (WHO 2002; DeMartino et al. 2017; Yue et al. 2020). Also, it can be used as an additive to the drilling mud to increase the efficiency of the hydraulic fracturing extraction of unconventional oil and gas (WHO 2002; Rich et al. 2016). However, CS_2_ is considered a toxic chemical; it seriously impacts the environment and public health (Rhodes et al. 2000; Rich et al. 2016; Saleh 2020; Printemps et al. 2022). CS_2_ can be released to the atmosphere due to biological activities and anthropogenic actions such as burning fuel (petroleum, gas, coal) containing CS_2_ (Bocos-Bintintan and Ratiu 2020). In atmosphere, CS_2_ is the most important volatile sulfur components that are responsible for the presence of sulfate aerosols in the stratospheric layer (Lennartz et al. 2020). Aso, CS_2_ is considered an indirect greenhouse gas, converted to CO_2_, consequently increasing its amount in the atmosphere (Montero-Campillo et al. 2018). To meet the UN’s sustainable development goals (SDG 7 and 13) (UN 2015) for providing a clean source of energy and accelerating climate change mitigation, CS_2_ must be removed during fuel processing.

Sulfur components can be removed from the fuels by different processes such as catalytic (hydro-or oxidative), biological, absorption by physical sorbents, or adsorption desulfurization (Speight 2011; Hsu and Robinson 2017; Sadare et al. 2017; Saleh 2020). It must be noted that the removal of the hydrogen sulfide (which is the major sulfur compound in the fuel) does not guarantee the removal of CS_2_ (Dan et al. 2012). This is because it is much less acidic than H_2_S, so conventional H_2_S removal methods, such as physical solvents, do not effectively remove the CS_2_ (Kohl and Nielsen 1997).

Among the desulfurization methods, we focused on adsorption desulfurization due to its advantages. It is economically viable; it can be performed at mild temperature and pressure conditions, the sulfur component can be recovered and utilized, and the adsorbent can be regenerated and reused (Chen et al. 2017; Iruretagoyena and Montesano 2018). Several adsorbents such as modified zeolites, metal–organic framework (MOF), activated carbon, metal oxides, e.g., Cu, Fe, Zn), etc., have been reported (Ma et al. 2005; Guo et al. 2006; Chen et al. 2017; Iruretagoyena and Montesano 2018; Georgiadis et al. 2020; Wang et al. 2021a; Hernández-Fernández et al. 2022).

Activated carbon is one of the most widely used adsorbents for pollutant removal, including gaseous and liquid contaminants. Coal, peat, wood, and various waste biomass are examples of carbonaceous substances employed as carbon precursors (Haggag et al. 2021). Date stone biomass contributes significantly to agricultural waste despite having little commercial value. According to the FAO, Egypt is also the world’s top producer of dates (El-Sharabasy and Rizk 2019). Date stone usage as a carbon source is economically advantageous (Ebiad et al. 2020). The biomass-derived materials have several applications environmental, wastewater treatment, climate change mitigation, and soil health improvement. Thus, biomass utilization is a way to achieve the UN sustainability goals (Wang et al. 2022).

Zinc oxide has been reported previously as a desulfurization adsorbent at medium to high temperatures (Frilund et al. 2020; Georgiadis et al. 2020). Also, it was reported that CS_2_ could react with primary and secondary amines (Kohl and Nielsen 1997). Zinc oxide can be synthesized by a homogeneous precipitation process using urea hydrolysis (Table S1) (Bitenc et al. 2008; Padmanabhan et al. 2009; Alhawi et al. 2015; Mantovani et al. 2017). Our previous studies indicated that controlling the urea hydrolysis conditions results in the insertion of nitrogen-containing anions (NH_2_CO^−^, isocyanate, or cyanate) within the structure of the adsorbent (Sakr et al. 2013, 2018, 2021).

In this work, we aimed to remove CS_2_ from the gasoline fraction using zinc hydroxide loaded on the surface of carbon material produced from biomass as an adsorbent. We focused on these materials due to the following features:

- The date stone biomass is considered a renewable feedstock for carbon (Mehmandoust et al. 2023).
- Zinc-based materials and carbon-derived date stones are reported to be low toxic and biodegradable (Zhang et al. 2013; Moustafa et al. 2018; Verma et al. 2021; Fan et al. 2022). After adsorption, if happened with carbon disulfide, the presence of a disulfide group in both zinc and carbon materials enhances their biodegradability and lowers their toxicity (Li et al. 2014; Onwudiwe et al. 2016; Martín et al. 2019; Saiyed et al. 2021).
- The adsorbent under investigation could be regenerated by heating (~ 100 ℃) under a flow of nitrogen, under a flow of steam, or boiling water (Yang et al. 2006; Wang et al. 2015; McGuirk et al. 2018).
- Even though the spent adsorbent is becoming inactive, it can be treated properly and optimized to produce a valuable product such as biogas (Chen et al. 2020; Wang et al. 2021b.

We synthesized zinc-carbon composite in situ using homogeneous precipitation of zinc hydroxide by controlled urea hydrolysis with the assistance of microwave irradiation (as a green source of energy) (Baghbanzadeh et al. 2011). These anions may affect CS_2_ adsorption. The CS_2_ removal was studied using a batch adsorption system at low temperature and atmospheric pressure. To the best of our knowledge, there is no reported data considering the loading of Zn-based material on carbon surfaces using controlled urea hydrolysis. Also, there is no reported data about using this composite as an adsorbent of CS_2_ from gasoline fraction (Tables S2 and 2).

## Material and methods

The chemicals used are zinc nitrate, hexahydrate (Zn(NO_3_)_2_.6H_2_O) (assay ≥ 99%), and urea (assay = 99%) purchased from Sigma-Aldrich. Ammonium hydroxide are from Caledon Laboratories and heptane from CARLO ERBA. All chemicals are used without any further purification. The water was distilled and then deionized using LABCONCO, Water Pro (USA) deionizer.

### Material synthesis

Zinc materials were prepared either by conventional/homogenous precipitation pathways. The pH meter model pH-213 was used to measure the changes in pH in all of the synthesis reactions (Hanna, USA).

#### Synthesis of zinc hydroxide by conventional precipitation method

To a solution containing zinc nitrate (0.05 M), ammonia solution (0.5 M) was added dropwise until the white precipitate was formed. The final pH reached 7.15. The precipitate (ppt) was then collected, centrifuged using MPW-352, Poland, and washed with deionized water several times. Then it dried in an oven at 80 °C.

#### Synthesis of zinc hydroxide by homogenous precipitation method

This synthesis protocol is similar to our previous work (Sakr et al. 2018). In a typical synthesis, a solution containing zinc nitrate (0.05 M) and urea (0.5 M) was subjected to microwave irradiation (180 W) in a domestic microwave oven for 90 min. The temperature reached 95 °C after 10 min and was constant along the reaction time. The synthesis reaction was done in an open glass vessel under atmospheric pressure. After the time for synthesis, the reaction was terminated immediately by cooling it down. As in step 1, the white-formed ppt was centrifuged, washed, and dried.

#### Synthesis of carbonized date stones

The carbon was prepared from date stones, and the detailed synthesis method was described (Ebiad et al. 2020). The typical synthesis cleaned date stones (washed with distilled water) were dried at 105 °C and sieved from 1 to 2 mm. Then it is placed in a quartz tube inside a horizontal tube furnace (Nabertherm, Labothem Model R50/250/12; Germany) and heated up to 600 °C under nitrogen flow (100 mL/min) for 3 h. The obtained carbon was then ground and sieved.

#### Synthesis of zinc-carbon composite

In a glass container, 2 g of the carbonized date stones was added to the solution containing zinc nitrate (0.05 M) and urea (0.5 M), then subjected to microwave irradiation. The same procedure was applied as in step 2 to compare the results. A gray ppt is formed, collected, and centrifuged; washed several times with deionized water; and dried at 80 °C. For simplicity, samples were coded as indicated in Table 1.

**Table 1:** The sample codes for the prepared adsorbents

Sample code	Material	Synthesis method	Precipitating agent	Heating source	Synthesis temperature	Final pH
Z	Zinc hydroxide	Conventional precipitation	Ammonium hydroxide	––	Room temperature	7.15
ZU	Zinc hydroxide	Homogenous precipitation	Urea	M.W	95 ℃	6.22
ZC	Zinc-carbon composite	Homogenous precipitation	Urea	M.W	95 ℃	6.01
C	Carbonized date stones	Calcination of date stones	–-	Horizontal tube furnace	600 ℃	–-

### Characterization

The crystalline structures of the synthesized solids were analyzed by X-ray diffraction (XRD) (X Pert PRO, PANalytical, the Netherlands) using Ni-filtered Cu Kα radiation operated at 40 kV. The spectra were recorded in an angular region of 2*Ɵ* = 4–80° with a step size at 2*Ɵ* = 0.02° and a scanning step time of 0.6 s.

The prepared adsorbents’ Fourier transform infrared (FT-IR) spectra were analyzed using a Nicolet IS 50FTIR Spectrometer (Thermo-Fisher, USA). Each adsorbent was diluted with potassium bromide (KBr) and compressed in the form of a thin disc and subjected to IR irradiation. The spectral wavelength region was from 4000 to 400 cm^−1^.

The surface textural properties of the prepared adsorbents were characterized using nitrogen adsorption/desorption isotherm data obtained at 77 K (NOVA, Quantachrome Instruments).

The surface morphology of the prepared adsorbents was examined using field emission scanning electron microscope (Carl ZEISS, sigma VP 300). The instrument also allows energy-dispersive spectroscopy (EDS) using the Zeiss SmartEDX detector.

### Adsorption activity

The CS_2_ adsorption ability of the prepared adsorbents was tested using a batch reactor (60-mL closed glass tube). A known amount of adsorbent was placed, mixed with a known volume of model component (heptane), representing the gasoline faction containing CS_2_ with an initial concentration of 500 ppm. This mixture was stirred for 90 min (using a Thermo-scientific Stirrer, USA) at the required temperature. The CS_2_ concentration was analyzed before and after the adsorption process using gas chromatography–chemiluminescence detector (GC-SCD) instrument, Agilent Technology, USA. The analysis method is performed according to the ASTM D5623 (D5623 2004) standard method, which is specified for analyzing sulfur compounds in low boiling point petroleum fractions.

The effect of temperature (30, 50, and 60 °C) on the adsorption process for all adsorbents under investigation is tested. Its dosage effect is tested for the most active adsorbent (20, 40, 60, 80, and 100 mg). The effect of time is also examined (60, 90, 120, 180, 210, and 240 min) at a working temperature of 30 °C.

The adsorption capacity was calculated as follows (Swat et al. 2017; Ebiad et al. 2020):

1 $$q=\left(Co-C\right)\frac{V}{w}$$

*Co* (mg/L) and *C* (mg/L) are the initial and at equilibrium solution concentrations of CS_2_, respectively; *V* (L) is the volume of the solution; and *w* (g) represents the mass of adsorbents. The removal % (*ɳ*) can be calculated as follows:

2 $$\eta =(\frac{Co - C}{Co})100$$

### Adsorption kinetics

Two main kinetic model groups describe the adsorption reaction (Vareda 2023):

- Pseudo-first order and pseudo-second order are the two widely used kinetic models that could be applied to the experimental adsorption data to assess adsorption reaction kinetics. The pseudo-first-order model of adsorption’s differential form can be written as follows (Lagergren 1898):
  3 $$\frac{{dq}_{t}}{dt}={\mathrm{k}}_{1}({\mathrm{q}}_{\mathrm{e}}-{\mathrm{q}}_{\mathrm{t}})$$
  where *k*_1_ is the equilibrium constant (min^−1^), and *q*_e_ and *q*_t_ (mg.g^−1^) are the amounts of CS_2_ adsorbed at equilibrium and at time *t*, respectively. Using Eq. (3)’s integration and the initial conditions *q*_t_ = 0 at *t* = 0,
  4 $$\log\left(q_{\mathrm e1}-q_{\mathrm t}\right)=\log\;q_{\mathrm e1}-(\frac{k_1}{2.303})t$$
- The pseudo-second-order reaction equation’s differential version can be expressed as (Ho and Mckay 1999):
  5 $$\frac{{dq}_{t}}{dt}={k}_{2}({q}_{\mathrm{e}}-{q}_{\mathrm{t}}{)}^{2}$$
  where *k*_2_ (mg.g^−1^.min^−1^) is the pseudo-second rate constant. The linearized form of this model is produced after integration, taking the boundary conditions into account as follows:
  6 $$\frac{t}{{q}_{\mathrm{t}}}=\frac{t}{{q}_{\mathrm{e}2}}+\frac{1}{{k}_{2}{q}_{\mathrm{e}2}^{2}}$$

### Mechanism of adsorption

Based on the kinetic data CS_2_ diffusion during the adsorption process could be predicted using the following models.

#### Intraparticle diffusion model

Weber and Morris are the first to descript the intraparticle diffusion model, where the rate-controlling step is due to the intraparticle diffusion (Weber and Morris 1963), where the adsorbate uptake during adsorption was proportional to the square root of the contact time:

7 $${q}_{\mathrm{t}}= {K}_{id }{t}^{0.5} + {C}_{i}$$

*K*_id_ is the intraparticle diffusion rate constant [mg.g^−1^ (min^0.5^)^−1^]. While *C* is the intercept, the value of *K*_id_ is determined by the slope of the straight line. The thickness of the boundary layer is evaluated by the value of *C*. The boundary layer effect increases with increasing intercept *C*.

#### Boyd’s film-diffusion model

This model assumes that the layer surrounding the adsorbent particle is responsible for resistance to adsorbate diffusion. The Boyd kinetic equation (Boyd et al. 1947) is denoted as

8 $$F\left(\mathrm{t}\right)=1-\frac{6}{{\pi }^{2}}\sum\nolimits_{n=1}^{\infty }\frac{{e}^{{-n}^{2}Bt}}{{n}^{2}}$$

where *F* is the fractional attainment of the equilibrium at a different time (*t*) and *B*(*t*) is a mathematical function of *F*.

9 $$F=\frac{{q}_{\mathrm{t}}}{{q}_{\mathrm{e}}}$$

where *q*_t_ and *q*_e_ are the amounts adsorbed at the time (*t*) and equilibrium, respectively.

Reichenberg was successful in getting the following estimates (Reichenberg 1953):

10 $$\mathrm{For}\;F\;\mathrm{values }>0.85\;B\left(t\right)=-0.4977-\mathrm{ln}\left(1-F\right)$$

11 $$\mathrm{And\;for}\;F\;\mathrm{values }< 0.85\;B (t)={\left(\sqrt{\pi }-\sqrt{\pi -\left(\frac{{\pi }^{2}F\left(t\right)}{3}\right)}\right)}^{2}$$

### Adsorption thermodynamics

Thermodynamic parameters such as Gibbs free energy (∆*G*^o^), entropy (∆*S*^o^), and enthalpy (∆*H*^o^) were calculated using the following equations:

12 $${K}_{d}=\frac{{C}_{s}}{{C}_{e}}$$

13 $$\Delta {G}^{o}=\Delta {H}^{o}-T\Delta {S}^{o}$$

14 $$ln\;{K}_{d}=\frac{\Delta S}{R}- \frac{\Delta H}{RT}$$

where *C*_e_ is the equilibrium concentration (mg.L^−1^) of CS_2_ in the solution, *K*_d_ is the adsorption distribution coefficient, and *C*_s_ is the quantity of CS_2_ adsorbed on the adsorbent surface per liter of the solution at equilibrium. *R* is the gas constant and *T* is the temperature. The slope and intercept of Van’t Hoff plots of (ln *K*_d_) vs. 1/*T* were used to derive ∆*H*^o^ and ∆*S*^o^.

## Results and discussion

### pH change monitoring

The changes in the pH during the synthesis reaction of the Z.U. and ZC samples are discussed in detail in Section S1, Table S3, and Figs. S1 and S2 in the supplementary file. Under MW irradiation, the urea hydrolysis reaction is affected by the presence of carbon particles in the synthesis mixture (Figs. S1 and S2). Also, the final pH is higher in the absence of the carbon sample. This may indicate that the released OH^−^ is consumed to neutralize the acid sites in the carbon surface as well as precipitate the zinc hydroxide.

It was reported that urea decomposes in aqueous media when subjected to heating (Shaw and Bordeaux 1955; Fernández et al. 2009) according to the following equation:

15 $$\begin{array}{cc}({\mathrm{NH}}_{2}{)}_{2}\mathrm{CO}+3{\mathrm{H}}_{2}\mathrm{O}& \to \end{array}{\mathrm{HCO}}_{{3}^{-}}+{\mathrm{OH}}^{-} +2{\mathrm{NH}}_{{4}^{+}}$$

The release of the hydroxyl groups during the hydrolysis process is responsible for the precipitation of the Zn^2+^ ions in the form of zinc hydroxide or carbonate (Zhang and Li 2003). However, according to the synthesis conditions, several intermediate anionic groups could be formed, which in the end affects the structural features such as carbamates, cyanates, isocyanates, and carbonates (Saber and Tagaya 2005; Kloprogge et al. 2006; Mavis and Akinc 2006; Sakr et al. 2013, 2018, 2021; Faramawy et al. 2018)**.**

### XRD analysis

**Fig. 1:**
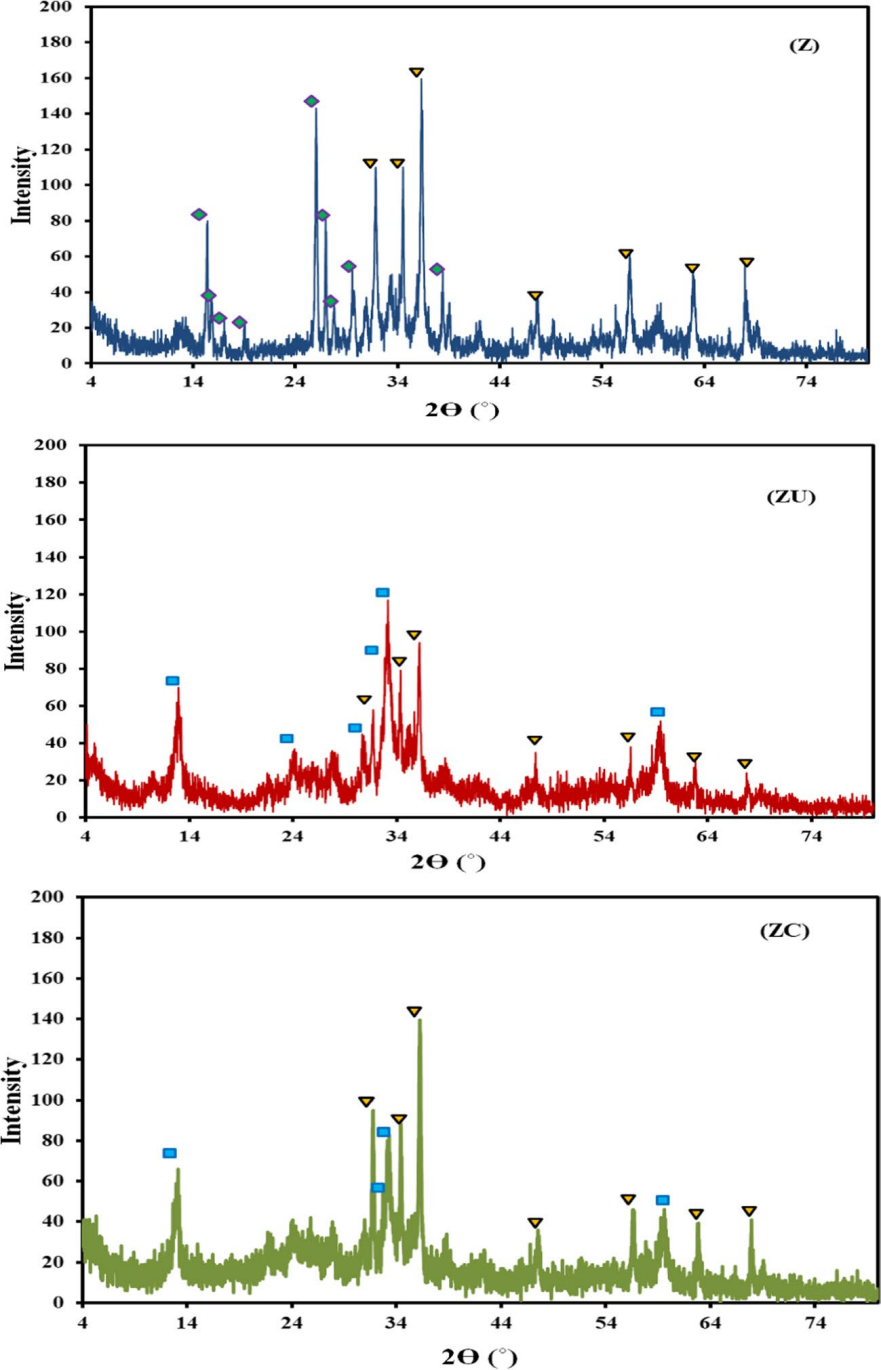
XRD patterns for (Z), (ZU), and (ZC) materials. The shape (  ) represents the zinc hydroxide (β-Zn(OH)_2_) phase, (  ) indicates the würtzite ZnO phase, and (  ) for the hydrozinicite phase

The XRD patterns of the prepared samples are represented in Figs. 1 and S3. The XRD pattern for the (C) sample (Fig. S3) shows the presence of two broad diffraction peaks at around 23.19 and 44.41 2*Ɵ*°, which correspond to the reflections of the (002) and (100) planes, respectively. The broadening and small intensity of the (002) plane may indicate the low degree of orientation of the aromatic layer in the three-dimensional aromatic carbon arrangement, while the broadening in the (001) plane may be related to the small aromatic layer slice in the carbon material (Qiu et al. 2019). This pattern indicates the presence of amorphous carbon with a low graphitization degree (Ebiad et al. 2020; Liu et al. 2021).

As indicated in Fig. 1, the Z sample (prepared conventionally) exhibits the diffraction peaks at 15.40, 15.87, 17.02, 18.99, 25.99, 26.96, and 27.76 2*Ө*°, revealing the presence of zinc hydroxide as compared to the zinc hydroxide (β-Zn(OH)_2_) reference (JCPDS 20–1435). The diffraction peaks detected at 31.8, 34.6, 36.4, and 47.7 2*Ө*° correspond to ZnO as compared to the reference pattern (JCPDS 05–0664) of ZnO.

The XRD pattern for the ZU sample (papered by homogenous precipitation) shows the presence of diffraction peaks at 12.99, 24.03, 27.88, and 33.14 2*Ө*° corresponding to hydrozinicite phase (zinc hydroxide carbonate, (JCPDS 14–0256)). In addition, a minor amount of ZnO diffraction peaks is also observed with diffident peak intensities compared to the **Z** sample. The low-intensity peak at 10.63 2*Ө*° could be a result of the existence of intercalated anion other than carbonate (Sakr et al. 2013).

For the composite sample (ZC), the XRD pattern resamples that of the ZU sample and reveals the hydrozinicite phase (zinc hydroxide carbonate (JCPDS 14–0256) and ZnO (JCPDS 05–0664), respectively). The main difference was the relatively high intensity of the peak at 36.23 2*Ө*° corresponding to the (101) phase. This may suggest that the presence of carbon material in the precipitation media stimulates the formation of the ZnO phase with different aspect ratios. The Zn oxide/hydroxide carbonate species may have uniformly covered the carbon material’s surface in the C sample, as evidenced by the loss of the amorphous carbon’s distinctive peak.

### FTIR spectra

**Fig. 2:**
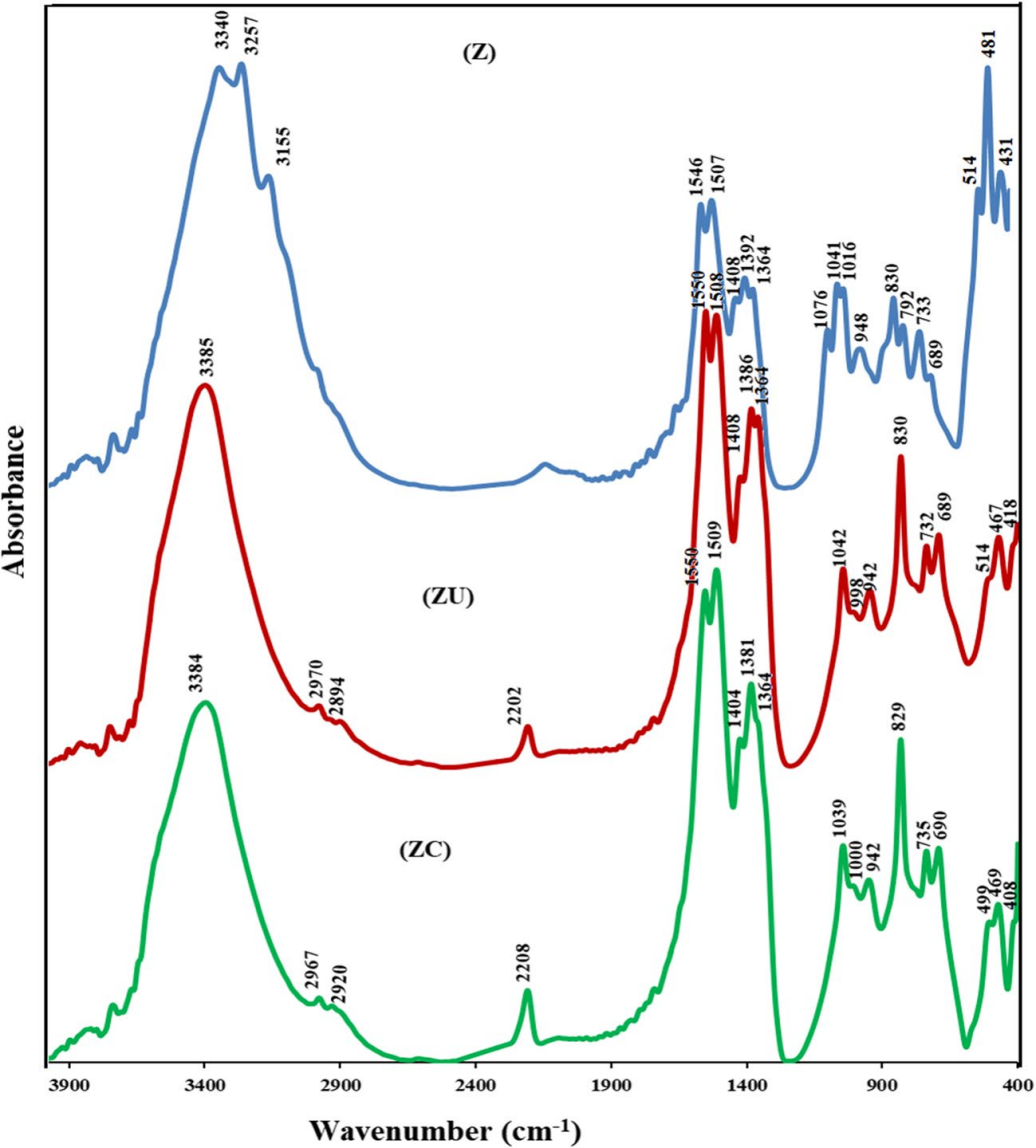
FTIR spectra for Z, ZU, and ZC materials

The structural vibrational region in the Z sample (Fig. 2) shows an absorption band at 481 cm^−1^, corresponding to the Zn–O bond stretching vibration in ZnO nanorods (Bundit and Wongsaprom 2018). The presence of the split peaks 514 and 431 cm^−1^ indicates the diversity of the particle morphology (Verges et al. 1990). A broad band in the region 3000–4000 cm^−1^ corresponds to the hydrogen-bonded hydroxyl groups. Bands at 1507 cm^−1^ and 1392 cm^−1^ (with the shoulder at 1363 cm^−1^) correspond to the vibration of hydroxyl groups bonded to Zn atoms and water (Giannakoudakis et al. 2015). The presence of 1363 cm^−1^ could result from C = O vibration from adsorbed CO_2_ on the surface. The band at 1041 cm^−1^ is assigned to Zn–OH bending vibration. The OH deformation band is detected at 830 cm^−1^ (Giannakoudakis et al. 2015) (Sec. S2).

The IR spectrum for the ZU sample is represented in Fig. 2. Two absorption bands appeared in the structural vibrational region 415 cm^−1^ and 467 cm^−1^ which could be attributed to the starching vibrational mode of the octahedral Zn_o_–O cluster and the Zn–OH translation vibration in the hydrozincite structure, respectively (Kloprogge et al. 2004; Gordeeva et al. 2020). The bands at 1550, 1386, and 732 cm^−1^ could correspond to the vibration modes of carbonate groups. The presence of a split at 1364 cm^−1^ could be due to the different modes of symmetric vibration of carbonate anion (bidentate) (Padmanabhan et al. 2009; Sakr et al. 2018). The broad band centered at 3385 cm^−1^ indicates the presence of hydrogen-bonded adsorbed water molecules with surface hydroxyl groups. The spectrum also exhibits a small absorption band at 2202 cm^−1^, corresponding to the cyanate group’s presence. The cyanate group is formed due to incomplete urea hydrolysis under the reaction conditions (Sakr et al. 2013). The results indicate the formation of the hydrozincite phase as well as of the zinc oxide phase. These results are in agreement with that observed by Padmanabhan et al. (2009), who stated that an amorphous intermediated Zn(OH)_2_ could be formed and transformed into the ZnO as a result of the synthesis conditions (Padmanabhan et al. 2009).

The IR spectrum of the sample ZU resembles that of the ample ZC (Fig. 2). However, the structural vibration region shows a little shift in the bands 514 cm^−1^ and 418 cm^−1^ in sample Z to be 499 cm^−1^ and 408 cm^−1^ in sample ZC, which could indicate the presence of another particle morphology-like prism formation (Verges et al. 1990). The presence of carbonate anions is detected with the characteristic band at 1386 cm^−1^ and a small shoulder at 1364 cm^−1^ (compared to that of the Z.U. sample). This may indicate that the monodentate carbonate anions present on the composite surface are predominating. The presence of urea-derived anions is also detected in the form of a cyanate group with a peak centered at 2208 cm^−1^.

The FTIR results are in agreement with those obtained from the XRD data. Under the synthesis conditions, the formed composite contains the zinc oxide/hydroxide carbonate with the presence of urea-derived anions as well.

### Field emission scanning electron microscope (FESEM) images

**Fig. 3:**
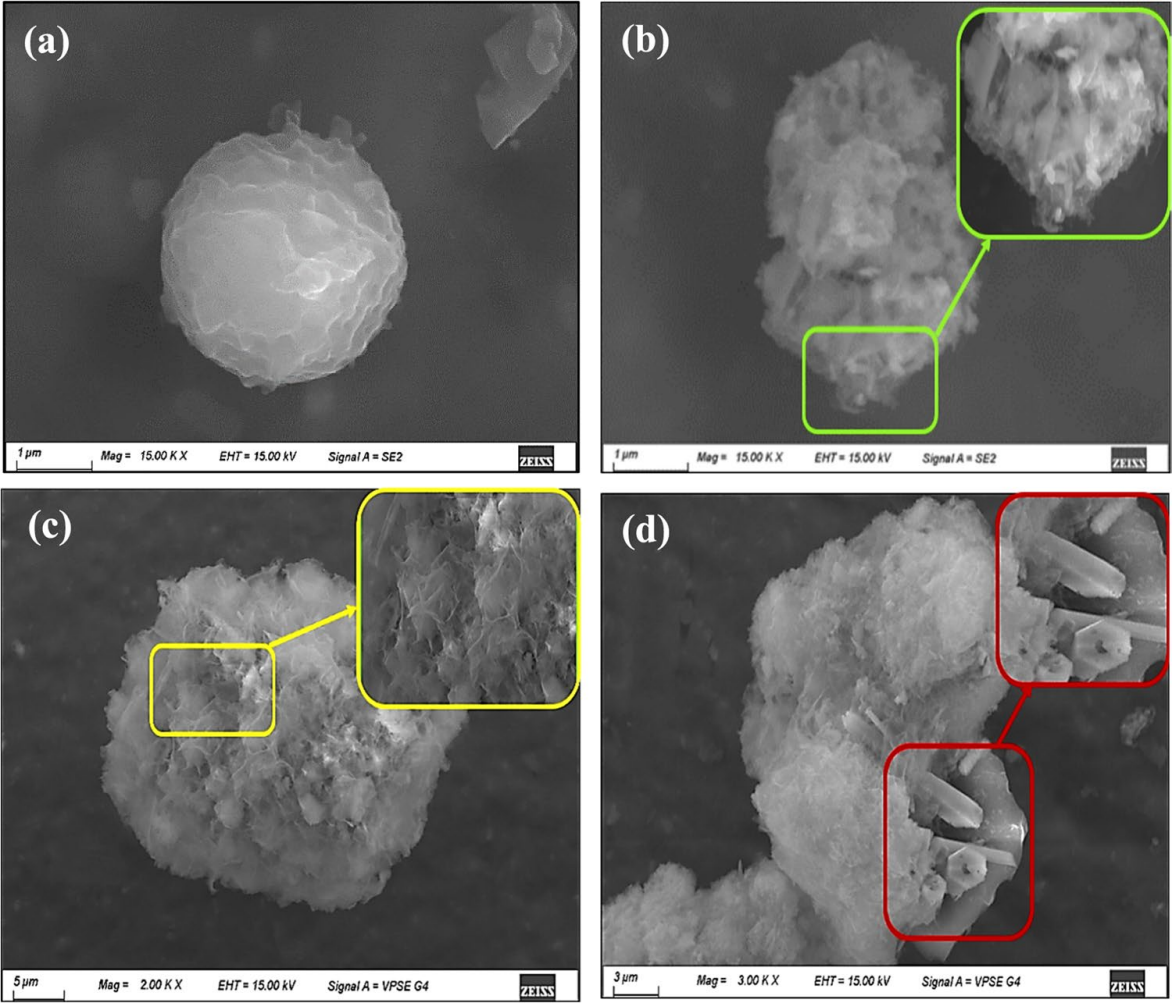
FESEM images for **a** C, **b**, Z, **c** ZU, and **d** ZC materials

The morphology of the prepared solids is shown in the FESEM images represented in Fig. 3. The C material shows irregular aggregates of stacked sheets (Fig. 3a). The Z material shows the formation of semispherical and rode-like particles (Fig. 3b), whereas the ZU sample images (Fig. 3c) indicate the presence of flakey-like particles (Padmanabhan et al. 2009) aggregated in large spherical particles. This is consistent with Molefe et al. (2015), who stated that temperature could act as a structural directing agent to gather the sphere-like particle to form a larger flak-like one (Molefe et al. 2015).

The particles of the ZC sample appeared in the form of a prism shape as well as flaky-like particles that coated the carbon particles (Fig. 3d). These data are confirmed from the EDS analysis of the ZC sample (Fig. S5), which reveals the formation of the Zn-carbon composite. The data from the FESEM images agree with those obtained from the XRD and FTIR results.

### Surface textural properties

**Fig. 4:**
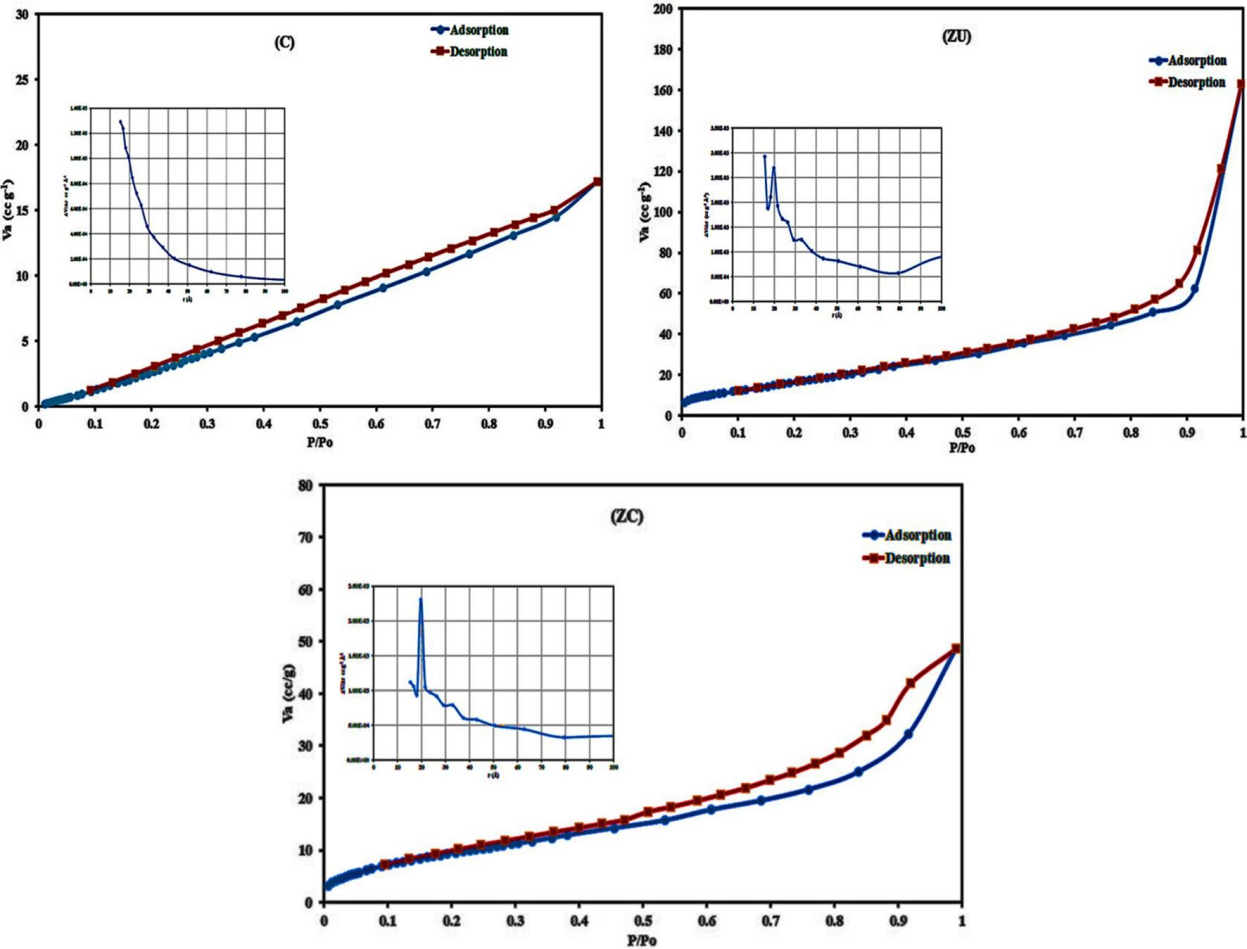
The N_2_ adsorption–desorption isotherm for C, ZU, and ZC materials. The insert figures indicate the BJH pore size distribution corresponding to each material

The textural characteristics of the prepared materials were tested using the nitrogen adsorption–desorption isotherm at low-temperature (Figs. 4 and S6 and Table S4). The specific surface area was calculated according to Brunauer–Emmett–Teller (BET) method. The pore size distribution and pore volume were calculated from the desorption curve in the isotherm using the Barrett-Joyner-Halenda (BJH) model.

The isotherm of the C sample reveals the presence of type III isotherm (according to the International Union of Pure and Applied Chemistry (IUPAC) classification), which indicates the presence of silt-like pores formed from the aggregation of plate-like particles (Ramimoghadam et al. 2013). The hysteresis indicates the presence of some mesoporosity that may be formed due to the aggregation of the particles. The BET surface area of the C sample was 26.89 m^2^ g^−1^. After loading with zinc hydro(oxide) particles, the BET surface area is slightly increased to 35.64 m^2^ g^−1^, which could be due to the C particle acting as a nucleus that helps the formation of a web or network from the Zn hydro(oxide) particles on its surface (Seredych et al. 2012; Giannakoudakis and Bandosz 2014). The surface area of the ZC is intermediate between that of the C and ZU samples, indicating the Zn material’s loading on the C surface (Mantovani et al. 2017) and confirming that obtained from the FE-SEM results. The isotherm of ZC samples is type IV with H3 hysteresis, which indicates mesoporosity due to the aggregation of the formed layered particles (Guo et al. 2016).

The BJH model was used to calculate the average pore size distribution (PZD) results, which show that the C sample has a PZD of < 15.33 Å, while the ZU sample has two modes of the pore size distribution (< 15.37 and 19.77 Å). The ZC sample possesses a narrow PZD of 19.75 Å. All the prepared solids show a PZD in the mesopore range which gives them an advantage in the adsorption of organic pollutants (Han et al. 2006).

### Adsorption activity

**Fig. 5:**
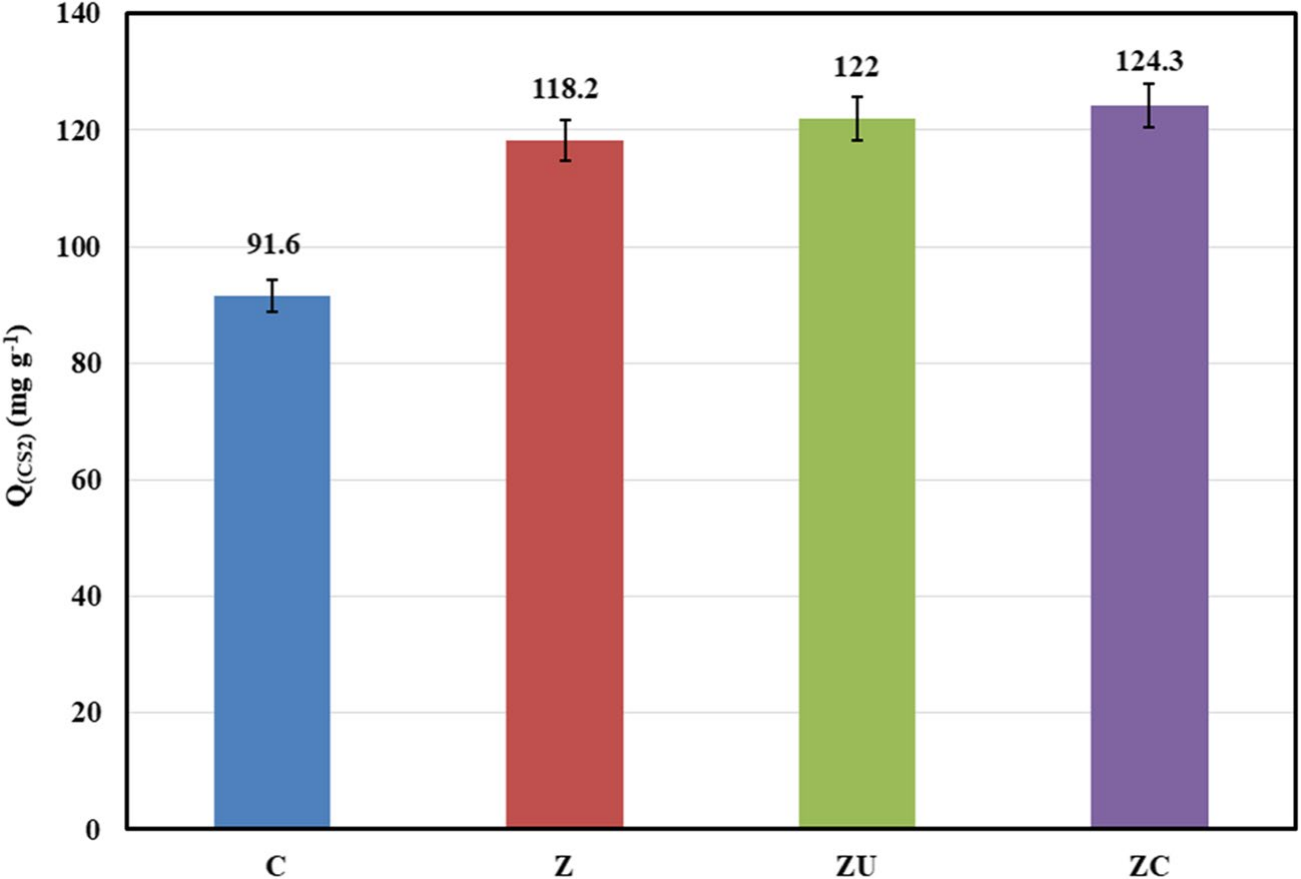
CS_2_ adsorption capacity diagram at 30 °C for the tested adsorbents

The adsorption process was carried out using a batch reactor at atmospheric pressure. The adsorption capacity and removal % (*ɳ*) were calculated using Eqs. (1) and (2), respectively. The CS_2_ adsorption capacities are shown in Fig. 5 at 30 °C and a constant weight of 20 mg. The data reveal that the adsorbents for capturing CS_2_ from the gasoline model component follow the order of C (91.6) < Z (118.2) < Zu (122) < ZC (124.3 mg (CS_2_)/g (adsorbent)).

The maximum adsorption capacity was found to be by the ZC adsorbent with an adsorption capacity of 124.3 mg (CS_2_)/g (adsorbent) with 49.7%. The obtained data is higher than reported in the literature using the adsorption technique at low temperatures (Table 2).

**Table 2:** Previously reported data on CS_2_ adsorption by activated carbon

Type of material	Reaction	Adsorption capacity	Temperature	Source	Reference
Zinc-carbon composite	Batch reactor	124.3 mg of CS_2_/g	30 °C	Hydrocarbon	This work
Cu/CoSPc/Ce modified activated carbon (AC_Cu‑CoSPc‑Ce_)	Fixed-bed quartz reactor system	Adsorption capacity of 17.39 mg of CS_2_/(g of activated carbon)	20 °C	Gas	(Wang et al. 2014)
Activated carbons	Batch system	The adsorption capacity of CS_2_ in damp gas is 60%–80% less than that in dry gas	50 °C	Gas	(Wang et al. 2011)
Active carbon fiber (ACF)	Batch system	The adsorption capacity of ACF is more extensive (72–104%) than that of GAC	150 °C	Water	(Yang et al. 2006)
Ion-exchanged zeolites Y	Fixed-bed adsorption column	The highest CS_2_ breakthrough adsorption capacity up to 44.8 mg/g	20 °C	Air	(Chen et al. 2017)
Polyacrylonitrile (PAN)-based activated carbon fiber (ACF)	A fixed-bed glass reactor	The best breakthrough adsorption capacity of CS_2_ was 55.63 mgS/g when CO activated the ACF	Room temperature	N_2_ gas	(Li et al. 2020)
Hydrophobization of activated carbon fiber (ACF) using vinyltrimethoxysilane	Glass vacuum system	The adsorption selectivity is improved under humid conditions	25 ℃	N_2_ gas in dynamic conditions	(Xie et al. 2011)
Activated carbon modified with KOH and ethylenediamine	Glass vacuum system	The CS_2_ adsorption is improved	30–60 ℃/0–30,000 Pa	–	(Guo et al. 2006)

**Scheme 1:**
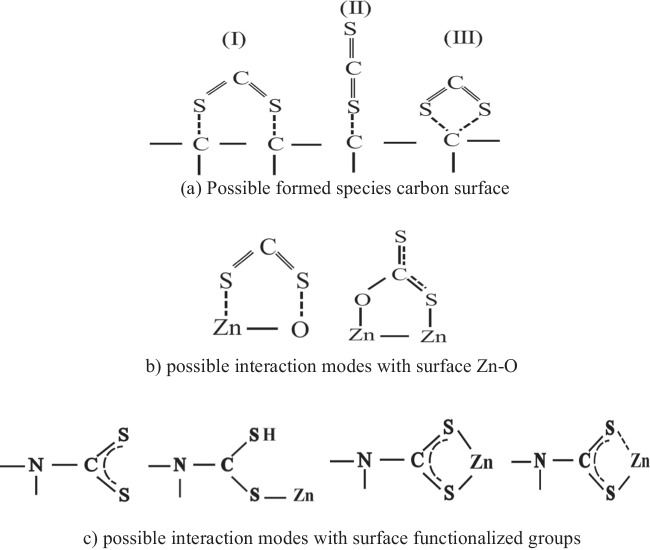
Possible accessible sites for CS_2_ adsorption on the ZC surface

This higher reactivity could be due to the surface texture of the prepared Zn-carbon composite, where the basic surface nitrogen species are formed during the urea hydrolysis and confirmed by the IR and XRD. This conclusion is supported by those reported previously (Kohl and Nielsen 1997; Guo et al. 2006; McGuirk et al. 2018; Orhan et al. 2019; Cao et al. 2020), where the presence of a nitrogen-containing group enhances the CS_2_ adsorption. In addition, the presence of the hydroxycarbonate group on the surface due to the urea hydrolysis reaction contributes to the CS_2_ adsorption (Kowalik et al. 2020). Also, CS_2_ can be physically adsorbed on the ZnO surface (Sahibed-Dine et al. 2000). In this work, the morphology of the ZnO oxide species with a prism shape on the surface of carbon particles in the ZC adsorbent may positively affect the CS_2_ adsorption process. This is in agreement with Ghenaatian and co-workers, who confirmed that the structural morphology of the ZnO particles plays an important role in the CS_2_ capture and storage process **(**Ghenaatian et al. 2013). Thus, according to the surface texture of the composite, the possible accessible active sites on the composite surface could be Scheme 1:

- At the carbon surface, CS_2_ could interact with the carbon atoms on the surface forming monodentate or bidentate interactions (Scheme 1a). The interaction, in this case, is considered very weak and CS_2_ capture is dependent mainly on the carbon porous structure (Yang et al. 2006).
- It can bind to the oxygen present in the ZnO crystal forming the carbonate which could be adsorbed by mono- or bidentate interaction, Scheme 1b (Sahibed-Dine et al. 2000).
- Carbon disulfide could interact with the nitrogen species that are present on the composite surface as a result of the controlled urea hydrolysis, forming thiocarbamate species (DeMartino et al. 2017; McGuirk et al. 2018). Thiocarbobamtes could be bound to the Zn by different intercalation modes (monodentate or bidentate), Scheme 1c (Saiyed et al. 2021).

**Fig. 6:**
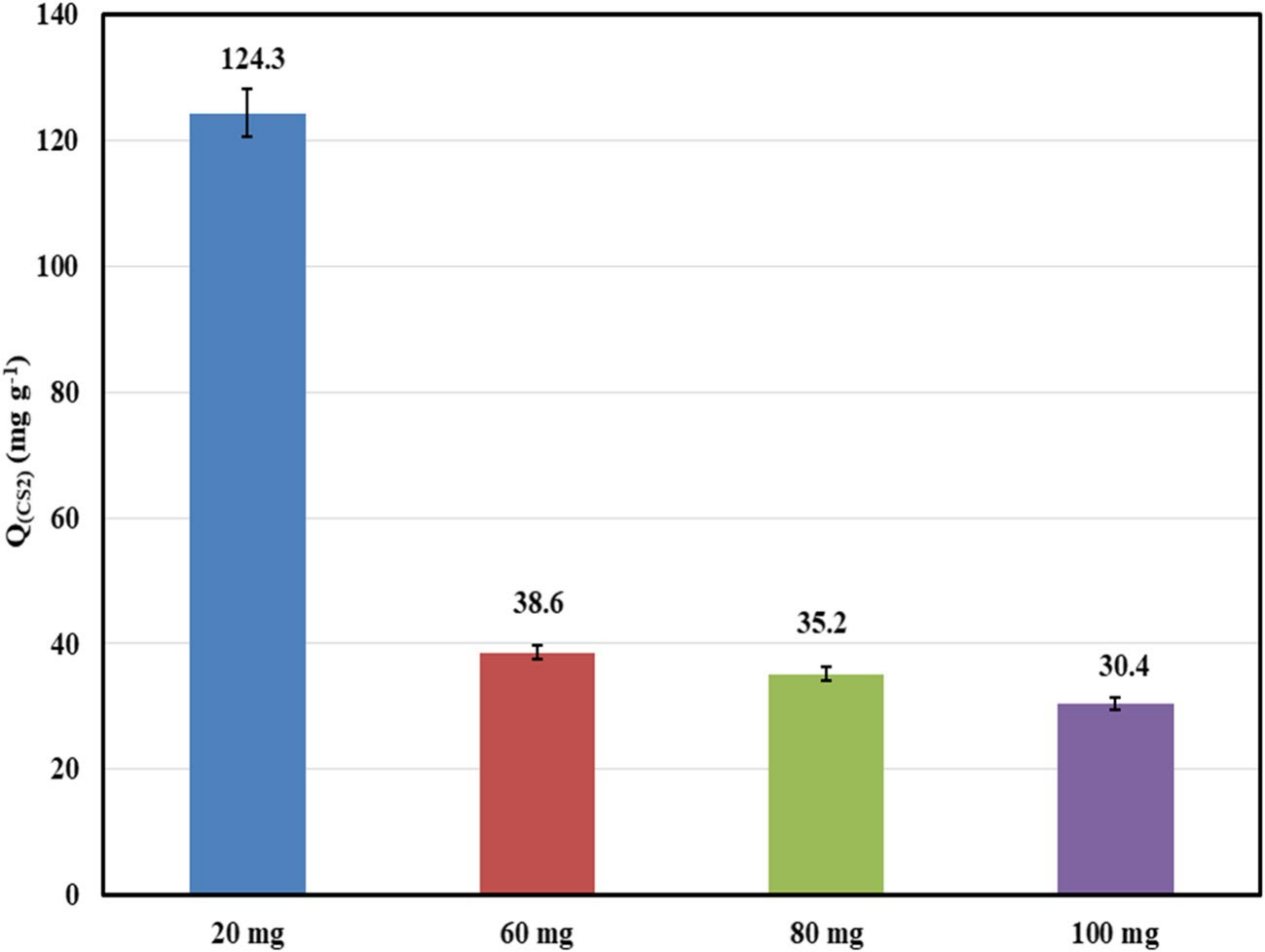
Effect of ZC adsorbent dose on the CS_2_ adsorption capacity

Figure 6 depicts the effect of the adsorbent dose on the adsorption process at 30 °C. It was observed that the adsorption capacity decreased with increasing the mass of the adsorbent. This may be due to the aggregation and accumulation of the adsorbent particles, which could hinder the active site of the adsorbent, making it less accessible to the CS_2_ molecules (Wang et al. 2010, 2015).

### Adsorption kinetics

**Fig. 7:**
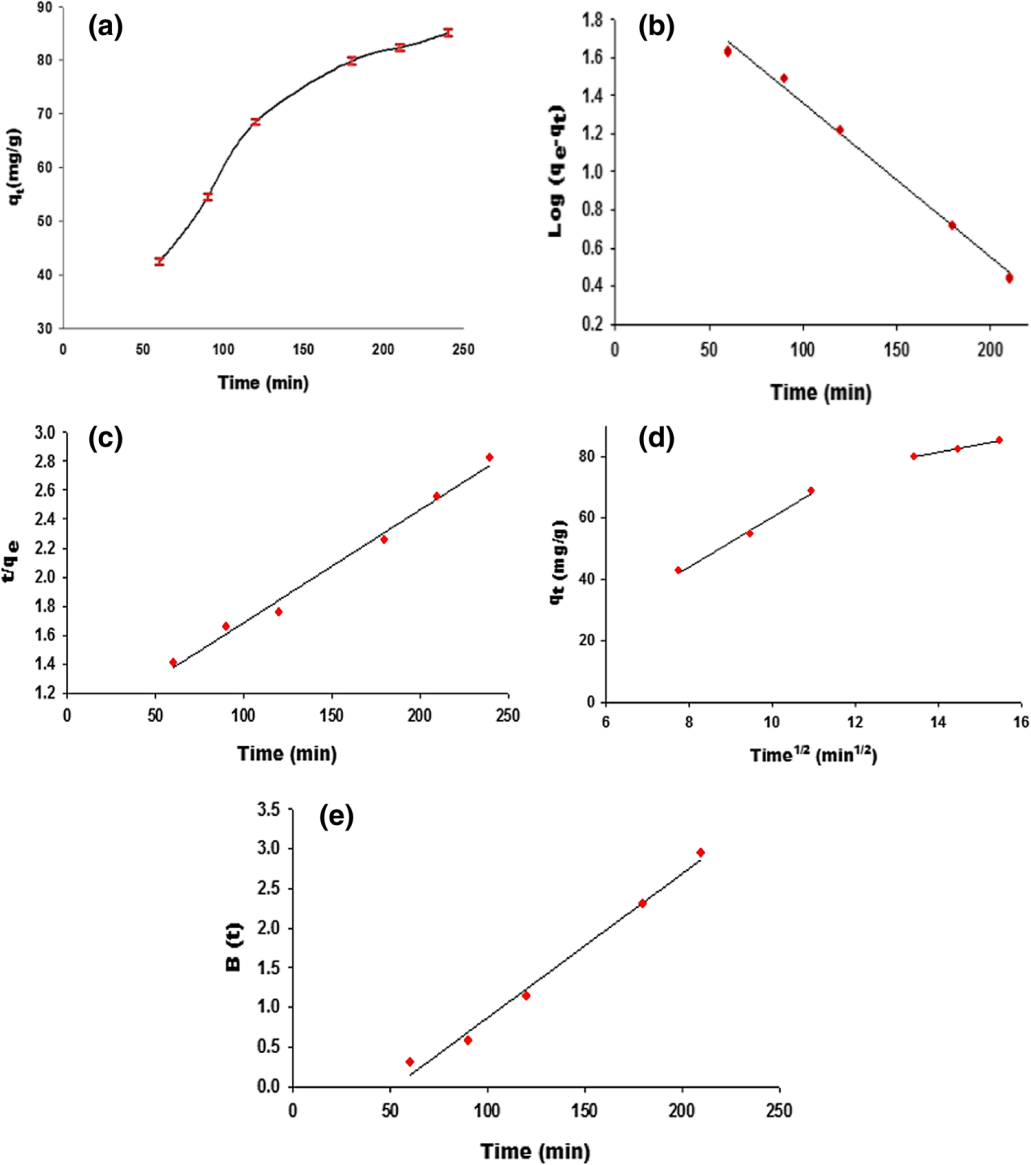
**a** Effect of time on CS_2_ adsorption by ZC sample at 30 °C, **b** pseudo-first-order kinetic model for adsorption, **c** pseudo-second-order kinetic model, **d** intraparticle diffusion plots, and **e**)Boyd plots for CS_2_ adsorption

We studied the kinetic behavior of CS_2_ adsorption onto ZC adsorbent at a working temperature of 30 °C and atmospheric pressure, considering the effect of time on the adsorption process (Fig. 7a). The CS_2_ adsorption increased quickly at first with time, then slowed down until equilibrium (which is not reached obtained under the experimental conditions). This increase may be due to the high concentration of CS_2_ and free active sites on the adsorbent surface between 0 and 120 min. Following that time, the number of available free active sites on the adsorbent surface became limited, resulting in a gradual decrease in the adsorption process.

Studying the adsorption process kinetics indicates its efficiency and applicability to process scaling up (Doǧan et al. 2009). The pseudo-first-order and pseudo-second-order kinetic models are applied to describe the adsorption reaction (“Adsorption kinetics” section; Eqs. (3)–(6)). The first model could predict the adsorption reaction through the adsorption rate on the adsorbent surface, and the second could predict the adsorption mechanism (Ebelegi et al. 2020).

From the results listed in Table 3 and Fig. 7(b and c), the pseudo-second-order (PSO) kinetic model offers the best agreement between the estimated values of *q*_e2_ and the experimental *q*_e_ data, with a high correlation coefficient of 0.9895. These findings imply that the PSO kinetic model was followed by the CS_2_ adsorption process on the ZC adsorbent. This alludes to the fact that chemisorption, which involves valence forces through sharing (covalent force) or exchange of electrons between sorbent and sorbate, regulates the adsorption process (Haggag et al. 2021). These results may reflect the role of the active sites, including the nitrogen-containing anions and Zn-species loaded on the carbon surface during the CS_2_ adsorption process.

**Table 3:** Kinetic parameters for the adsorption of CS_2_ onto CZ sample at 30 °C

Model	Constant parameter	30 °C
	*q*_e, exp_ (mg.g^−1^)	85.096
Pseudo-first-order	*q*_e1_ (mg.g^−1^)	8.758
	*k*_1_ (L.min^−1^)	0.0187
	*R*^2^	0.9737
Pseudo-second-order	*q*_e2_ (mg.g^−1^)	128.205
	*k*_2_ (g. mg^−1^. min^−1^)	2.78E − 5
	*R*^2^	0.9895
Intraparticle diffusion	*K*_ip_	8.044
	*C*	− 20.39
	*R*^2^	0.9909
Boyd plot	Intercept	− 0.9394
	*R*^2^	0.9896

#### Mechanism of adsorption

Three steps are typically used to illustrate the adsorption mechanism based on the kinetic data (Wu et al. 2009; Loganathan et al. 2014; Youssef et al. 2014): (i) film diffusion is the transfer of adsorbate molecules from the main body of the solution to the adsorbent’s surface; (ii) ions are moved from the surface to the intraparticle active sites (particle diffusion); and (iii) ions are adsorbed by the adsorbent’s active sites. The third step does not fall within the rate-controlling phases because it is a relatively quick process. Therefore, either film diffusion or particle diffusion is primarily responsible for the rate-controlling stages. Weber and Morris model (intraparticle diffusion model) is described in the “Mechanism of adsorption” section (Eq. (7)). Table 3 provides the results of the kinetic parameter variables *K*_id_, *C*, and *R*^2^.

The dual linear regions of this curve, according to this concept, can be attributed to the different adsorption extents at the beginning and final stages. The second region section rises gradually with the intraparticle diffusion, while the first steep represents the exterior surface adsorption. The plot of *q*_t_ vs. *t*^1/2^ should be linear and pass through the origin if intraparticle diffusion is the rate-limiting step. None of the intraparticle diffusion plots crossed through the origin, indicating that the intraparticle diffusion mechanism is not the only rate-controlling step and the film diffusion had an impact as well (boundary layer diffusion).

The kinetic data were subsequently examined using the Boyd kinetic model to discriminate between film diffusion and particle diffusion to forecast the slow step involved (“Mechanism of adsorption” section; Eqs. (8)–(11)) (Boyd et al. 1947; Loganathan et al. 2014). We investigate the linearity of the experimental value and the data listed in Table 3 by plotting *B*(*t*) against time *t*, as depicted in Fig. 7e. Particle-diffusion mechanisms govern the adsorption process if the plots are linear and pass through the origin. According to the findings, film diffusion governs the adsorption of CS_2_ on the ZC sample at 30 °C because the plot line does not pass through the origin (Fig. 7e) (Chen et al. 2010).

### Adsorption thermodynamics

**Fig. 8:**
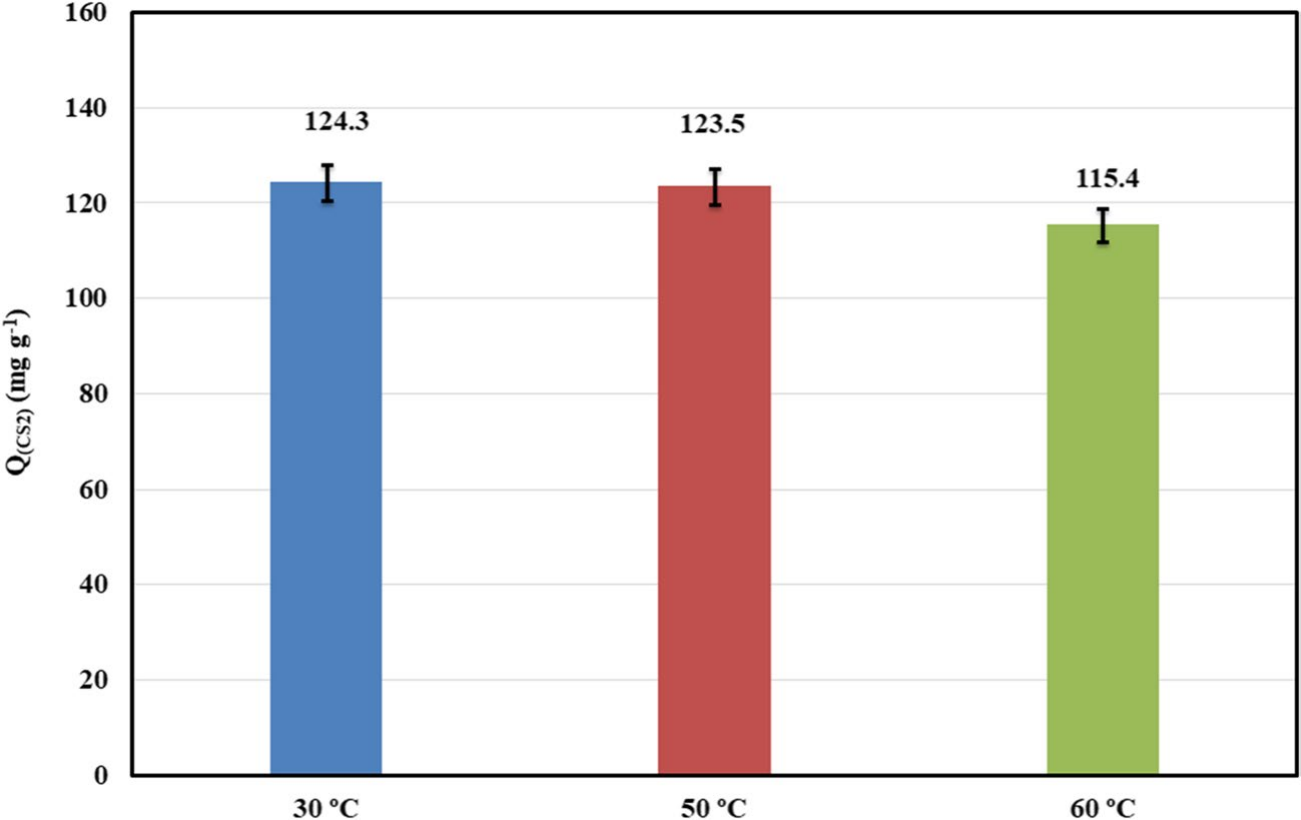
Effect of temperature on the CS_2_ adsorption capacity using ZC adsorbent

Figure 8 shows the effect of temperature on the CS_2_ adsorption capacity using ZC as an adsorbent with constant weight (20 mg) and atmospheric pressure. The data indicate that the capacity slightly decreased with increasing temperature in the physical adsorption process. The adsorption process is exothermic; consequently, it is favored at low temperatures (Wang et al. 2015). These results are assisted with the thermodynamic calculation (“Adsorption thermodynamics” section; Eqs. (12)–(14)). The thermodynamic consideration is important to evaluate the feasibility and spontaneity of the adsorption process (Ebelegi et al. 2020).

Table 4 lists the CS_2_ adsorption thermodynamic characteristics. The data reveals the negative ∆*G*° values which indicate the spontaneous adsorption of CS_2_ onto the ZC adsorbent. The adsorption of CS_2_ onto the ZC sample could be categorized as physisorption adsorption, with the change in free energy for this process ranging between − 2.14 and − 1.76 kJ.mol^−1^. It was reported that the ∆*G*° for chemisorption ranges between − 80 and – 400 kJ.mol^−1^ and that for physisorption ranges from − 20 to 0 kJ.mol^−1^ (Ebiad et al. 2020).

**Table 4:** Thermodynamic parameters for CS_2_ adsorption on ZC sample at 30 °C, 50 °C, and 60 °C

Sample	∆*H*° (kJ.mol^−1^)	∆*S*° (kJ.mol^−1^ K^−1^)	∆*G*°(kJ.mol^−1^)			*K*_d_		
			303 K	323 K	333 K	303 K	323 K	333 K
ZC	0.77	0.0116	− 1.76	− 0.89	− 2.14	2.012	2.024	2.166

The positive ∆*H*° values (1.77 kJ.mol^−1^) indicate that the adsorption of CS_2_ is endothermic. The ∆*S*° calculated positive values for ZC sample 0.0116 kJ.mole^−1^ K^−1^. These show an increase in unpredictability at the interface between the solid and the solution. To break through the activation energy barrier and increase the intraparticle diffusion rate, mobility must be increased. Based on the adsorption kinetic and thermodynamic results, it can be concluded that the CS_2_ adsorption on the Zn-carbon composite is a spontaneous and feasible process.

## Conclusion

In this study, the composite was formed from carbon-derived date stone biomass, and zinc hydroxide (ZC) was formed to be used as a CS_2_ adsorbent from the gasoline fraction. The loading of zinc hydroxide on the carbon surface was assisted by microwave irradiation using homogenous precipitation by urea hydrolysis. The study of the physicochemical characteristics shows the presence of flake-like zinc hydroxide particles formed on the carbon surface, making a net-like morphology. Prism-shaped zinc oxide particles were also detected, which were not detected in the sample zinc hydroxide prepared under the same conditions without carbon. This reflects the role of the presence of carbon particles in the synthesis reaction media.

The CS_2_ adsorption process from the gasoline fraction was done in a batch reactor at atmospheric pressure. The effects of temperature and the adsorbents’ dose on adsorption were examined. The best adsorption capacity (124.3 mg (CS_2_)/g) was for a zinc-carbon composite at 30 °C and atmospheric pressure compared with the parent materials (C, Z, and ZU). The obtained adsorption capacity was higher than that previously reported in the literature. The enhanced capacity is attributed to the ZC characteristics, where, Zn–O species, carbon active centers, urea-derived anions, formed zinc oxide particles, and composite surface texture properties could contribute to the CS_2_ adsorption process. These results highlight the efficiency of the proposed composite synthesis process and the role of urea-derived species in enhancing the adsorption capacity. The kinetics studies indicate that the adsorption process follows the pseudo-second-order kinetic model and that the adsorption mechanism is governed by film diffusion. From the obtained results, it can be concluded that the CS_2_ adsorption on the Zn–carbon composite is a spontaneous and feasible process.

## Supplementary Information

Below is the link to the electronic supplementary material.

Supplementary file1 (DOCX 555 KB)

## Data Availability

All related data and materials are within the manuscript.
