# Supplementary material for: Carbon disulfide removal from gasoline fraction using zinc-carbon composite synthesized using microwave-assisted homogenous precipitation
Source: Environ Sci Pollut Res Int. 2023 Jun 14;30(34):82014–30. doi: 10.1007/s11356-023-27905-2 (PMC10349739; doi:10.1007/s11356-023-27905-2)
Supplement: Supplementary file 1 — Supplementary file1 (DOCX 555 KB) [file 11356_2023_27905_MOESM1_ESM.docx]

(Supplementary File)

***Journal:***

**Environmental Science and Pollution Research**

**Title:**

**Carbon disulfide removal from gasoline fraction using zinc-carbon composite synthesized using microwave-assisted homogenous precipitation.**

Ayat A.-E. Sakr ^a.*^, Nouran Amr ^b^, Mohamed Bakry ^a,^*, Waleed I. M. El-Azab ^a^, Mohamed A. Ebiad ^a^

^a^  Analysis & Evaluation Division, Egyptian petroleum research institute, Nasr city, Cairo 11727, Egypt

^b^ Faculty of Biotechnology, October University for Modern Sciences and Arts (MSA), Egypt.

*. Corresponding authors:

(Ayat. A.-E. Sakr); Email: [ayatsakr@yahoo.com](mailto:ayatsakr@yahoo.com); [ayatsakr78@gmail.com](mailto:ayatsakr78@gmail.com); [ayatsakr@epri.sci.eg](mailto:ayatsakr@epri.sci.eg).

(Mohamed Bakry); Email: [moh.bakry81@yahoo.com](mailto:moh.bakry81@yahoo.com), [drmohamedbakry81@gmail.com](mailto:drmohamedbakry81@gmail.com)

[Mohamed.b.masod@epri.sci.eg](mailto:Mohamed.b.masod@epri.sci.eg)

| Content |  | page |
| --- | --- | --- |
| **Table S1** | **Some reported literature on the synthesis of zinc-based materials by urea hydrolysis** | **S3** |
| **Table S2** | **Previously reported synthesis conditions of Zn-carbon composites**. | **S4** |
| **Sec. S1** | **pH changes during the synthesis reaction**. | **S5** |
| **Table S3:** | **pH changes during the synthesis reaction of ZU and ZC samples.** | **S5** |
| **Figure S1:** | **pH changes of ZU sample during synthesis.** | **S6** |
| **Figure S2:** | **pH changes of ZC sample during synthesis**. | **S6** |
| **Figure S3** | **XRD pattern for (C) material.** | **S7** |
| **Figure S4** | **IR spectrum for (C) material.** | **S8** |
| **Figure S5** | **EDS analysis of the zinc-carbon composite material** | **S9** |
| **Figure S6** | **The N_2_ adsorption-desorption isotherm for Z material. The insert figures indicate the BJH pore size distribution.** | **S10** |
| **Table S4** | **Surface textural properties of the prepared adsorbents** | **S10** |
| **References** |  | **S11** |

Table S1: Some reported literature on the synthesis of zinc-based materials by urea hydrolysis.

| Material | Raw materials | Heating process | Reaction vessel | Temperature | References |
| --- | --- | --- | --- | --- | --- |
| ZnO micro-javelins | Zinc nitrate, urea | MW  (150 watt) | Teflon bomb |  | (Padmanabhan et al. 2009) |
| Hydrozincite | ZnCl_2_/ urea (1:5) | Oil bath | Three neck flask | 95 ℃ | (Mantovani et al. 2017) |
| Zinc hydroxide carbonate and porous zinc oxide | Zinc nitrate, urea (Zn^2+^: urea =1:5M) | Heating oven | Closed reactor (4hrs) | 85℃;  calcination at 600 ℃for 30 min | (Bitenc et al. 2008) |
| Zinc Carbonate Hydroxide | Zinc acetate dihydrate, urea (1:2) | Hydrothermal | Teflon-lined stainless steel autoclave | 120 ℃ | (Alhawi et al. 2015) |
| Zinc oxide/hydroxide carbonate | Zinc nitrate, urea (Zn^2+^: urea =1:10M) | Microwave irradiation (180 watt) at atmospheric pressure | Open glass vessel | 95 ℃ | This work |

Table S2: previously reported synthesis conditions of Zn-carbon composites.

| Composite | Carbon source | Zn-source/ precipitating agent | Process | Synthesis condition | Application | References |
| --- | --- | --- | --- | --- | --- | --- |
| Zinc(hydr)oxide/graphite | graphite-based | zinc chloride/ Sodium hydroxide | In situ-precipitation | Dried at 100 ℃ | Electrical conductivity | (Seredych et al. 2012) |
| zinc oxide/ reduced graphite oxide | Reduced graphite oxide | Zinc acetate/ Na Sodium hydroxide | Reduction by microwave | Freeze and vacuum dried | H_2_S gas dynamic adsorption at 300C | (Song et al. 2014) |
| Graphene oxide/Zinc oxide | graphene oxide nanosheets | ZnCl_2_ / NaOH | hydrothermal treatment at 180 C | Dried at 60 ℃ | Adsorption of methylene blue (MB) and methyl orange (MO) from an aqueous solution | (Archana et al. 2018) |
| graphene/zinc oxide | Modified graphite oxide | Zn(CH3COO)_2_.2H2O | The mixture was ground in a mortar and then placed in a glass vial | domestic microwave oven at 1000 watt. | supercapacitor performance | (Guo et al. 2016) |
| carbon/zinc oxide | poly(styrene-r-acrylonitrile) (PSAN) copolymers | zinc 2- ethyl hexanoate (Zn(EH)2 | Pyrolysis | Tube furnace at 800 ℃ | Pseudo-capacitor electrodes | (Zhao et al. 2018) |
| Zinc-carbon composite | date stone biomass | Zinc nitrate/ urea | In-situ homogenous precipitation/ microwave irradiation | 180 watt/ 95 ℃ | CS_2_ removal from gasoline fraction | This work |

**Sec (S1)**

**pH changes during synthesis reaction:**

During the synthesis reaction time, the pH changes mentoring the synthesis reaction were illustrated in **Table (S3)**, and Figures **(S1 and S2)**. For the ZU sample, nearly constant pH values were achieved for around 30 min of the MW time. Then a gradual increase in the pH up to 5.94 for another 40 min. The final region was a sharp increase to 6.22 at around10 min.

The pH changes for the ZC sample were different. The pH was gradually decreased to 4.7 in the first 20 min which could be attributed to the consumption of OH ions produced from the urea hydrolysis process (Zhang and Li 2003). Then it sharply increased to a value of 5.39 for the next 10 minutes. The pH final region was accompanied by a gradual increase in the next 50 minutes to 6.01.

These results indicate the effect of the carbon materials inside the synthesis reaction media. The noticed decrease (Region 1) in the ZC sample may be related to the neutralization of the carbon surface acid groups with the produced OH groups from urea hydrolysis. At the same time, Zn hydroxide could be formed on the carbon surface. This process affects the urea hydrolysis process and influences the final pH values which were observed to be less than that of the ZU sample. In contrast to that reported by Padmanabhan et al., (2009) who observed the precipitate after pH 6.11. In this work, the ppt is observed after pH 5.39 for both ZU and ZC samples. This indicates that the reaction conditions are highly influencing the resulting materials(Padmanabhan et al. 2009).

Table S3: pH changes during the synthesis reaction of ZU and ZC samples.

| *Material* | *Region1* | *Region 2* | *Region3* | *Final pH* |
| --- | --- | --- | --- | --- |
| ZU | 5.32 – 5.39 (plateau) | 5.39-5.94 (gradual increase) | 5.94-6.22 (sharp increase | 6.22 |
| ZC | 5.32-4.7 (gradual decrease) | 4.7-5.39 (sharp increase) | 5.39-6.01 (gradual increase) | 6.01 |

| 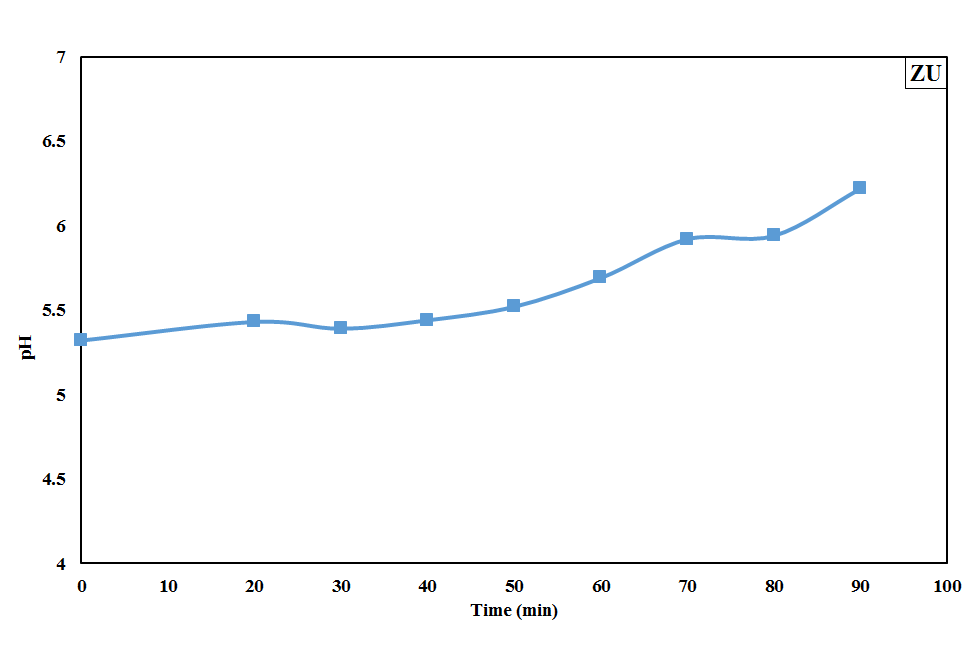 |
| --- |
| Figure (S1): pH changes of ZU sample during synthesis. |

| 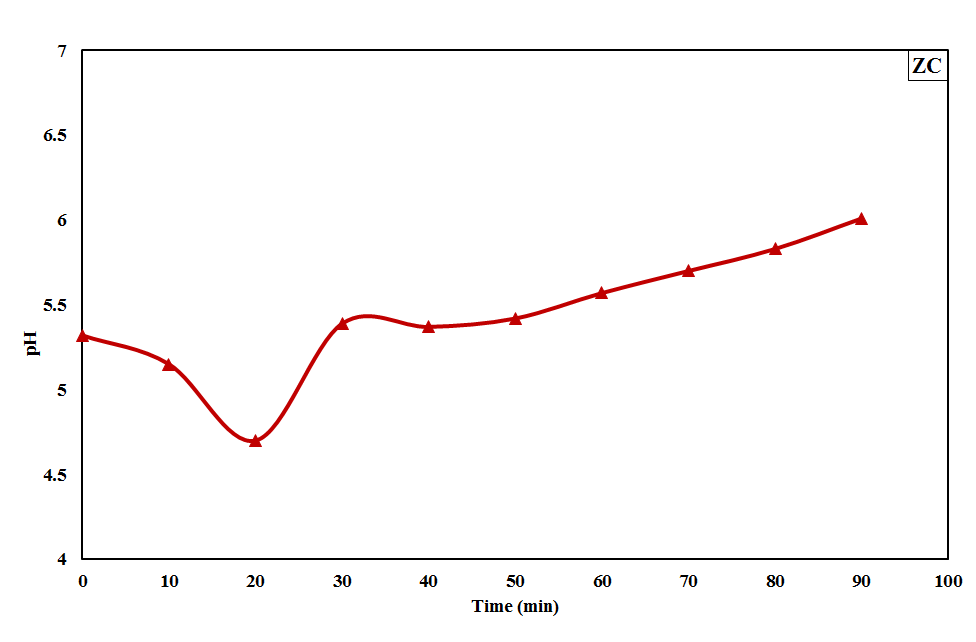 |
| --- |
| Figure (S2): pH changes of ZC sample during synthesis. |

| 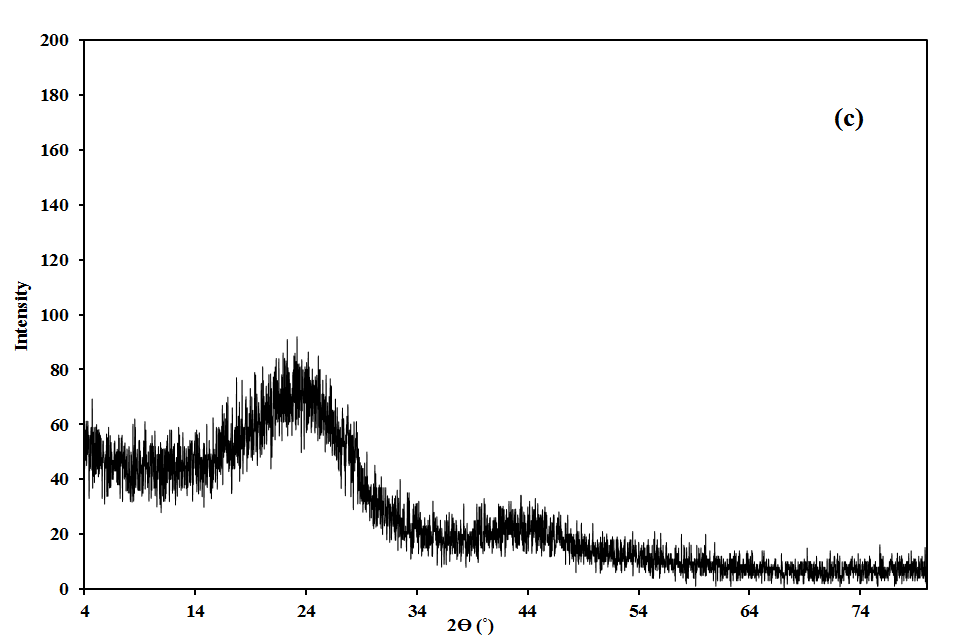 |
| --- |
| Figure (S3): XRD pattern for (C) material. |

| 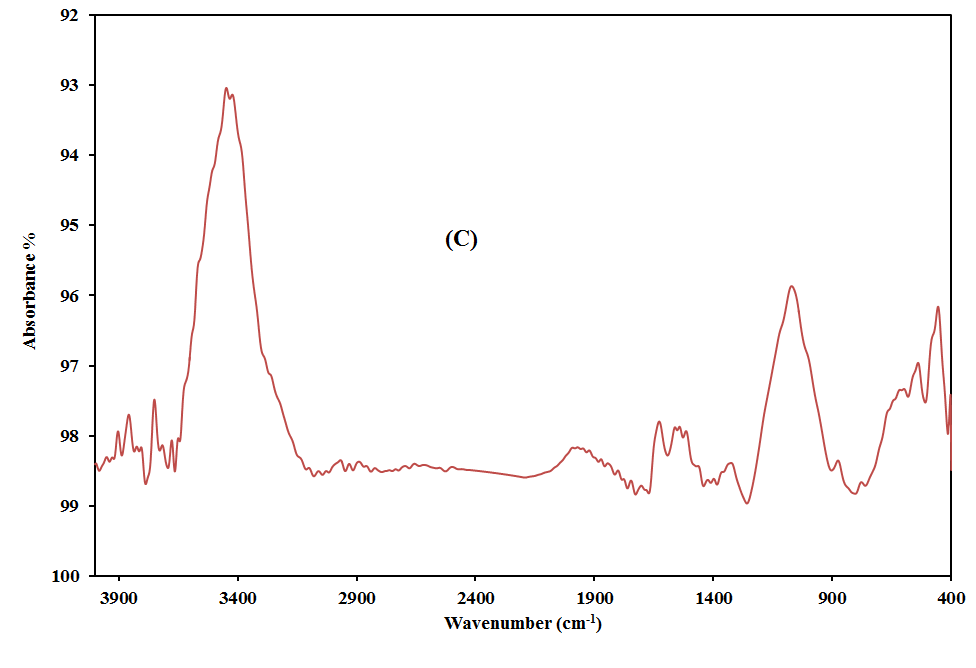 |
| --- |
| Figure (S4): IR spectrum for (C) material. |

| 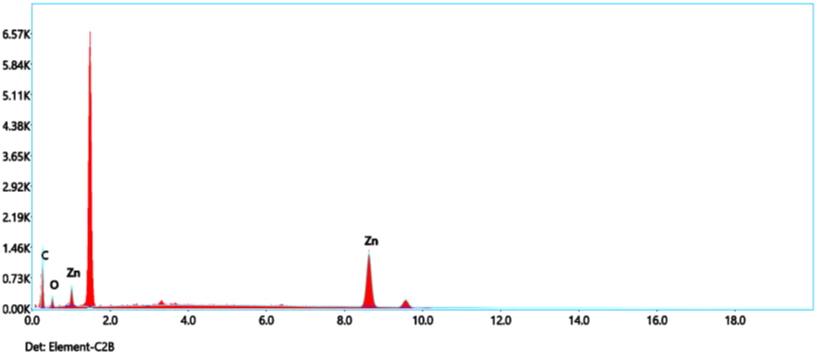 |
| --- |
| Figure S5: EDS analysis of the zinc-carbon composite material. |

| 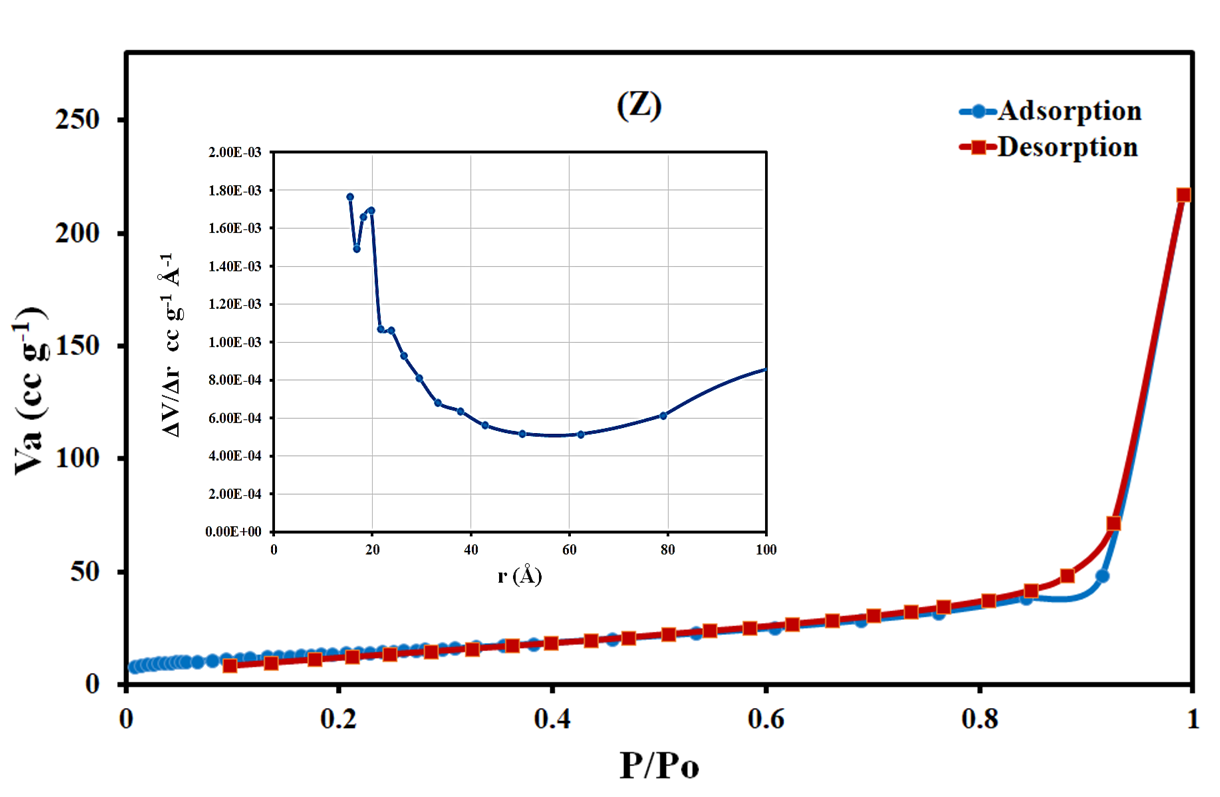 |
| --- |
| Figure S6: The N_2_ adsorption-desorption isotherm for Z material. The insert figures indicate the BJH pore size distribution. |

Table S4: Surface textural properties of the prepared adsorbents.

| *Sample name* | *S_BET_ (m^2^ g^-1^)* | *Vp (cc g^-1^)* | *(BJH) r_p_(Å)* |
| --- | --- | --- | --- |
| C | 26.89 | 0.02659 | <15.33 |
| ZU | 70.39 | 0.2519 | <15.37,  (19.77) |
| ZC | 35.64 | 0.07516 | 19.75 |
| Z | 50.49 | 0.3362 | <15.41,  (19.75) |

**References:**

Alhawi T, Rehan M, York D, Lai X (2015) Hydrothermal Synthesis of Zinc Carbonate Hydroxide Nanoparticles. Procedia Eng 102:356–361. https://doi.org/10.1016/j.proeng.2015.01.161

Archana S, Kumar KY, Jayanna BK, et al (2018) Versatile Graphene oxide decorated by star shaped Zinc oxide nanocomposites with superior adsorption capacity and antimicrobial activity. J Sci Adv Mater Devices 3:167–174. https://doi.org/10.1016/j.jsamd.2018.02.002

Bitenc M, Marinšek M, Crnjak Orel Z (2008) Preparation and characterization of zinc hydroxide carbonate and porous zinc oxide particles. J Eur Ceram Soc 28:2915–2921. https://doi.org/10.1016/j.jeurceramsoc.2008.05.003

Guo Y, Chang B, Wen T, et al (2016) One-pot synthesis of graphene/zinc oxide by microwave irradiation with enhanced supercapacitor performance. RSC Adv 6:19394–19403. https://doi.org/10.1039/c5ra24212f

Mantovani KM, Stival JF, Wypych F, et al (2017) Unusual catalytic activity after simultaneous immobilization of two metalloporphyrins on hydrozincite/nanocrystalline anatase. J Catal 352:442–451. https://doi.org/10.1016/j.jcat.2017.06.015

Padmanabhan SC, Ledwith D, Pillai SC, et al (2009) Microwave-assisted synthesis of ZnO micro-javelins. J Mater Chem 19:9250–9259. https://doi.org/10.1039/B912537J

Seredych M, Mabayoje O, Koleśnik MM, et al (2012) Zinc(hydr)oxide/graphite based-phase composites: effect of the carbonaceous phase on surface properties and enhancement in electrical conductivity. J Mater Chem 7970–7978. https://doi.org/10.1039/c2jm15350e

Song HS, Park MG, Ahn W, et al (2014) Enhanced adsorption of hydrogen sulfide and regeneration ability on the composites of zinc oxide with reduced graphite oxide. Chem Eng J 253:264–273. https://doi.org/10.1016/j.cej.2014.05.058

Zhang SC, Li XG (2003) Preparation of ZnO particles by precipitation transformation method and its inherent formation mechanisms. Colloids Surfaces A Physicochem Eng Asp 226:35–44. https://doi.org/10.1016/S0927-7757(03)00383-2

Zhao Y, Wang Z, Yuan R, et al (2018) ZnO/carbon hybrids derived from polymer nanocomposite precursor materials for pseudocapacitor electrodes with high cycling stability. Polymer (Guildf) 137:370–377. https://doi.org/10.1016/j.polymer.2018.01.024
